# The Arg99Gln Substitution in HNRNPC Is Associated with a Distinctive Clinical Phenotype Characterized by Facial Dysmorphism and Ocular and Cochlear Anomalies

**DOI:** 10.3390/genes16020176

**Published:** 2025-02-01

**Authors:** Luigi Chiriatti, Manuela Priolo, Roberta Onesimo, Mattia Carvetta, Chiara Leoni, Alessandro Bruselles, Francesca Clementina Radio, Camilla Cappelletti, Marco Ferilli, Daniela Ricci, Marcello Niceta, Viviana Cordeddu, Andrea Ciolfi, Cecilia Mancini, Giuseppe Zampino, Marco Tartaglia

**Affiliations:** 1 Molecular Genetics and Functional Genomics, Bambino Gesù Children’s Hospital, IRCCS, 00143 Rome, Italy; luigi.chiriatti@opbg.net (L.C.); mattia.carvetta@opbg.net (M.C.); fclementina.radio@opbg.net (F.C.R.); camilla.cappelletti@opbg.net (C.C.); marco.ferilli@opbg.net (M.F.); marcello.niceta@opbg.net (M.N.); andrea.ciolfi@opbg.net (A.C.); cecilia.mancini@opbg.net (C.M.); marco.tartaglia@opbg.net (M.T.); 2 Medical and Molecular Genetics, AORN A. Cardarelli, 80131 Naples, Italy; 3 Center for Rare Diseases and Birth Defects, Department of Woman and Child Health and Public Health, Fondazione Policlinico Universitario A. Gemelli, IRCCS, 00168 Rome, Italy; roberta.onesimo@policlinicogemelli.it (R.O.); chiara.leoni@policlinicogemelli.it (C.L.); 4 Department of Biochemical Sciences “Alessandro Rossi Fanelli”, Sapienza University, 00185 Rome, Italy; 5 Facoltà di Medicina e Chirurgia, Università Cattolica del Sacro Cuore, 00168 Rome, Italy; 6 Department of Oncology and Molecular Medicine, Istituto Superiore di Sanità, 00161 Rome, Italy; alessandro.bruselles@iss.it (A.B.); viviana.cordeddu@iss.it (V.C.); 7 Department of Biomedicine and Prevention, University “Tor Vergata”, 00173 Rome, Italy; 8 Department of Computer, Control and Management Engineering, Sapienza University, 00185 Rome, Italy; 9 National Centre of Services and Research for Prevention of Blindness and Rehabilitation of Low Vision Patients, IAPB Italia Onlus, 00185 Rome, Italy; d.ricci@iapb.it; 10 Pediatric Neuropsychiatric Unit, Department of Woman and Child Health and Public Health, Fondazione Policlinico Universitario A. Gemelli, IRCCS, 00168 Rome, Italy

**Keywords:** *HNRNPC*, MRD74, phenotypic heterogeneity, CHARGE syndrome, cochlear aplasia, coloboma, microphthalmia, genotype-phenotype correlations

## Abstract

*Background/Objectives:* Heterozygous variants in the heterogeneous nuclear ribonucleoprotein C gene (*HNRNPC*) have recently been reported to cause intellectual developmental disorder-74 (MRD74), a neurodevelopmental disorder with no recurrent diagnostic handles. Affected individuals show variable, non-specific, and subtle dysmorphic features. The degree of developmental delay (DD)/intellectual disability (ID) is also wide, ranging from mild to severe. The mutational spectrum is relatively broad with exon deletions and splice site and frameshift variants distributed along the entire length of the gene leading to HNRNPC loss of function. Only two missense changes located within the RNA-binding motif (RBM) and adjacent linker region of the more abundant isoform (Arg64Trp and Arg99Gln) have been described. Notably, the Arg99Gln amino acid substitution was reported in a subject presenting with a more complex and unique clinical phenotype characterized by distinctive facial features, DD/ID, cochlear aplasia, and bilateral colobomatous microphthalmia, suggesting the possible occurrence of phenotypic heterogeneity. *Results:* Here, we report the second individual carrying the Arg99Gln change in HNRNPC and having clinical features with a significant overlap with the peculiar phenotype of the previously described subject, supporting the occurrence of a genotype–phenotype correlation. *Conclusions:* Due to the concomitant occurrence of ocular and cochlear involvement as recognizable diagnostic handles, we propose that the *HNRNPC*^Arg99Gln^-related phenotype should be considered as a potential differential diagnosis in subjects with ID and major signs of CHARGE syndrome not fulfilling the minimum criteria for a clinical diagnosis.

## 1. Introduction

Heterogeneous nuclear ribonucleoproteins (HNRNPs) are a large family of ubiquitously expressed RNA-binding proteins involved in gene expression and nucleic acid metabolism, including pre-mRNA processing, mRNA stabilization during export from the nucleus to the cytoplasm, and regulation of the translational process [1,2]. To date, genes encoding members of this family have been proven to contribute to various neurodegenerative conditions, including amyotrophic lateral sclerosis (*HNRNPA1*), oculopharyngeal muscular dystrophy (*HNRPA2B1*), spinal muscular atrophy (*HNRNPG*), and fronto-temporal lobe dementia (*HNRPA2B1*) [1]. Disease-causing variants encompassing eight genes encoding members of this family have consistently been associated with syndromic neurodevelopmental disorders (NDDs) (*HNRNPH1,* MIM 601035; *HNRNPH2*, MIM 300610; *HNRNPK,* MIM 600712; *HNRNPR,* MIM 607201; *HNRNPU*, MIM 602869; *HNRNPQ,* MIM 616686; *HNRNPG,* MIM 300199; *HNRNPC,* MIM 164020) [3,4,5,6,7,8,9,10,11,12]. Among them, heterozygous *HNRNPC* loss-of-function (LoF) variants have recently been identified to cause a syndromic NDD (i.e., intellectual developmental disorder, autosomal dominant 74, MRD74; MIM 620688), mainly characterized by global developmental delay (DD), intellectual disability (ID), behavioral abnormalities, and variable facial dysmorphism [11].

*HNRNPC* codes for the heterogeneous nuclear ribonucleoprotein C (HNRNPC), a key protein involved in pre-mRNA splicing by promoting alternative exon usage [13], and in the export of transcripts longer than 700 nucleotides from the nucleus to the cytoplasm [14]. HNRNPC is characterized by five functional domains: a conserved *N*-terminal single-stranded RNA-binding motif (RBM) previously known as the RNA-binding domain (RBD) (residues 1 to 93), a linker region conferring RNA-binding specificity for the poly r(U)_5_ consensus sequence (residues 94 to 104), a positively charged zipper-like motif (bZLM) (residues 140 to 179), a leucine-zipper-like oligomerization domain (CLZ) (residues 180 to 207), and a *C*-terminal domain (residues 208 to 290) with a still poorly characterized function [11,15,16]. HNRNPC’s target accessibility is regulated by the mRNA post-transcriptional modification N(6)-methyl-adenosine (m^6^A), a mechanism known as “m^6^A-switch” [17], regulating the alternative splicing, export, maturation, and expression of several target mRNAs. Notably, *HNRNPC* haploinsufficiency has been shown to affect these processes, leading to the nuclear accumulation of ID-associated mRNAs [11,17].

The phenotypic spectrum of MRD74 is typically non-specific. Affected individuals show variable and subtle dysmorphic features, with brachycephaly, deep-set eyes, either hypo- or hypertelorism, a prominent nose, a smooth philtrum, and a thin upper lip occurring most commonly [11]. DD/ID and behavioral anomalies are invariably present, with the former characterized by wide variability, ranging from mild to severe. A relatively broad spectrum of pathogenic variants leading to HNRNPC LoF have been reported, the majority representing nonsense, frameshift, and intragenic in-frame deletions. In addition, two functionally uncharacterized missense changes (Arg64Trp and Arg99Gln) located within the RBM and adjacent linker region of the more abundant HNRNPC isoform (isoform b, NP_004491.2) have also been described [11]. Notably, among the previously reported individuals, the subject carrying the Arg99Gln amino acid substitution presented with a more complex and atypical clinical phenotype, characterized by distinctive facial features, DD/ID, cochlear aplasia, and bilateral colobomatous microphthalmia, suggesting the possible occurrence of phenotypic heterogeneity [11]. Of note, cochlear hypoplasia and colobomatous microphthalmia represent two major signs of CHARGE syndrome according to the diagnostic criteria available for this disorder [18,19].

Here, we report a second individual carrying the same missense mutation in *HNRNPC* and showing an overlapping phenotype with the previously described subject, indicating the occurrence of a genotype–phenotype correlation. Although the clinical phenotype of the two subjects does not fulfill the minimum criteria for a clinical diagnosis of CHARGE syndrome [19], due to the co-presence of two major diagnostic features, we propose that HNRNPC^Arg99Gln^-related phenotype should be considered as a potential differential diagnosis in subjects with ID and major signs of CHARGE syndrome.

## 2. Materials and Methods

The subject was enrolled in a research program aiming to understand the molecular causes of unclassified pediatric disorders at the Ospedale Pediatrico Bambino Gesù, Rome, Italy. The study was approved by the local Institutional Ethical Committee (ref. RF-2021-12374963, 15 March 2023). Clinical data, pictures, and blood samples were collected, used, and stored after signed informed consent from the participating subject and her parents was secured, in accordance with the ethical standards outlined in the Declaration of Helsinki. Permission to publish the clinical pictures was obtained.

### 2.1. DNA Methylation Profiling Analysis

Peripheral blood (PB) DNA was extracted using standard techniques. Bisulfite conversion was performed, and samples were analyzed using the Infinium Methylation EPIC BeadChip v.1/v.2 (Illumina San Diego, CA, USA), according to the manufacturer’s protocol. IDAT files containing methylated and unmethylated signal intensities were imported for analysis into R v.4.4.0 by means of the minfi package, correcting for background intensities. DNA methylation (DNAm) analysis was performed using a previously described pipeline [20,21] and the currently available DNAm signature for CHARGE syndrome [22,23], and grouped by means of hierarchical clustering (HC) and multidimensional scaling (MDS), considering the pairwise Euclidean distances between samples. The training model of the support vector machine (SVM) machine learning (ML)-based classifier was carried out as previously described [24].

### 2.2. WGS Analysis

Trio-based WGS data were obtained using a 2×150 bp paired-end read protocol on a NovaSeq 6000 platform (Illumina, San Diego, CA, USA), reaching 30x median coverage. Base calling and data analysis were performed using Bcl2FASTQ (Illumina). Paired-end reads were mapped to the GRCh38 reference sequence; variant calling and joint genotyping were run using Sentieon v.2023-08 (https://www.sentieon.com). SNP and indel hard filtering were applied using Genome Analysis Toolkit, version 3.8.0 (Broad Institute). High-quality variants were first filtered by frequency ≤ 5% in the in-house WGS population-matched database (>350 WGS). The remaining coding sequence variants were annotated and filtered using a custom pipeline, as previously described [25,26]. Detected variants in non-coding regions were annotated and prioritized using Genomiser v.2309 [27]. Structural variants were detected using DELLY v.1.1.6 [28] and prioritized using AnnotSV v.3.4. [29]. The identified *HNRNPC* variant was validated by bidirectional Sanger sequencing.

### 2.3. Structural Analysis

The non-covalent intramolecular interactions involving Arg^99^ in the wild-type (WT) HNRNPC structure (PDB ID: 2MXY, and PDB ID: 2MZ1) [30] and the predicted structural consequences of the Arg99Gln amino acid change were inspected using UCSF Chimera software v.1.17.3 (https://www.cgl.ucsf.edu/chimera, accessed on 9 December 2024) [31].

## 3. Results

### 3.1. Clinical Findings

**Figure 1:**
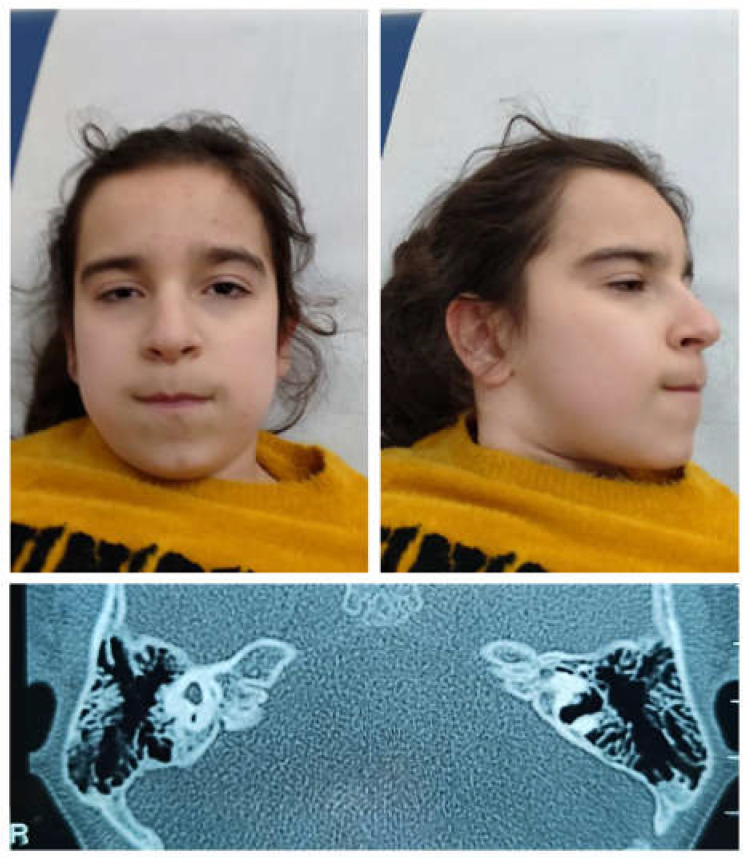
Clinical features of the subject carrying the missense *HNRNPC* pathogenic variant predicting the Arg99Gln amino acid substitution. Facial features (top panels). Note a broad and slightly receding forehead, high hairline, thick eyebrows, narrow and wave-shaped lids, bilateral microphthalmia, sharp nose, long philtrum, thin upper lip, prominent malar region, dimple chin, slightly posteriorly rotated ears, large triangular dimple, and fleshy lobes. CT scan of mastoids and petrous bone (bottom panel). Note aplasia of the left internal auditory canal (IAC), marked hypoplasia of the right IAC, and severe malformation of the vestibule, semicircular canals, and cochleae, bilaterally.

The proband was a single-born from an uneventful pregnancy of non-consanguineous parents without a family history of genetic or neurological diseases. The parents reported three previous spontaneous miscarriages during the first trimester. A cesarean section was performed because of the lack of progress in labor. Her auxological parameters at birth were as follows: weight, 3730 g (+1.37 SD); length, 52 cm (+1.61 SD); and occipitofrontal circumference (OFC), 36 cm (+1.87 SD). Her Apgar score was 8 and 9, at the first and fifth minute, respectively. She was fed with valid suction. At birth, a bilateral periauricolar fistula was noticed and auditory evoked potentials were performed, revealing hearing loss. At 1 year, brain and ear MRI was performed, which evidenced agenesis of the left cochlea, while a sketch of a cochlea was present on the right; therefore, acoustic prosthesization was prescribed. A CT scan was later performed, documenting the complex malformation of the inner ears (Figure 1). The ophthalmological evaluation showed iris, chorioretinal, and optic nerve coloboma with microphthalmia and subluxation of the crystalline lens on the right eye, and chorioretinal coloboma on the left eye. At the cardiological evaluation, a patent ductus arteriosus was documented. She underwent endocrinological evaluation because of growth retardation. Growth hormone (GH) stimulation tests ruled out GH deficiency. She presented with menarche at the age of 15 with irregular menses.

She presented with DD. At one year, she started rehabilitation. She achieved sitting at 18 months and independent walking at 30 months. A neuropsychological assessment documented moderate intellectual impairment (IQ 42). She currently attends high school with a support and communication assistant and use of alternative augmentative communication.

At the last evaluation (18 years), she presented with global growth delay: weight, 40.9 kg (−2.26 SD); height, 142.3 cm (−3.18 SD); and OFC, 50 cm (−4.05 SD). She presented with a broad and slightly receding forehead, a high hairline, thick eyebrows, narrow and wave-shaped palpebral fissures, bilateral microphthalmia with iris coloboma (right eye), a sharp nose, a thin upper lip, slightly posteriorly rotated ears with a fistula at the root of the helix bilaterally, a large triangular dimple, fleshy lobes, a prominent malar region, and a dimple chin (Figure 1). She also showed a short and broad distal phalanx of the first finger of the hands bilaterally.

Due to the presence of two major features of CHARGE syndrome, comparative genomic hybridization analysis and mutation scanning of the entire coding sequence of *CHD7* (MIM 608892) was performed, both resulting as negative.

### 3.2. Molecular Findings and Structural Analysis

To definitively exclude the suspicion of an atypical presentation of CHARGE syndrome, DNAm profiling was performed. A set of 148 differentially methylated probes defining the previously reported episignature for CHARGE syndrome [22,23] was used to test and classify the DNAm profile of the subject, which was compared with two in-house cohorts composed of three patients with molecularly confirmed CHARGE syndrome (ID1: NM_017780.4:c.2839C>T, p.Arg947Ter; ID2: NM_017780.4:c.7252C>T, p.Arg2418Ter; ID3: and NM_017780.4:c.1972G>T, p.Glu658Ter) (“CHD7 training set”) and 400 controls (290 healthy subjects and 110 individuals affected with different rare disorders). Unsupervised and supervised analyses consistently grouped the tested sample together with controls (Supplemental Figure S1), in line with the previous negative genetic findings.

A subsequent WGS analysis identified a de novo single-nucleotide variant (NM_031314.3:c.296G>A; NP_112604.2:p.Arg99Gln) in the *HNRNPC* gene. Variant validation and segregation were attained by Sanger sequencing (Supplemental Figure S2). A likely pathogenic impact of the amino acid substitution was predicted by the AlphaMissense (score = 0.94) and CADD (PHRED score = 29) tools. The c.296G>A change was not present in gnomAD v.4.1.0 but had been reported in the original cohort of MRD74 patients reported by Niggl et al. [11]. The WGS data analysis confirmed the absence of intragenic or structural variants involving *CHD7* and excluded the occurrence of other clinically relevant variants in genes previously reported to be implicated in neurodevelopmental disorders with features overlapping with CHARGE syndrome.

**Figure 2:**
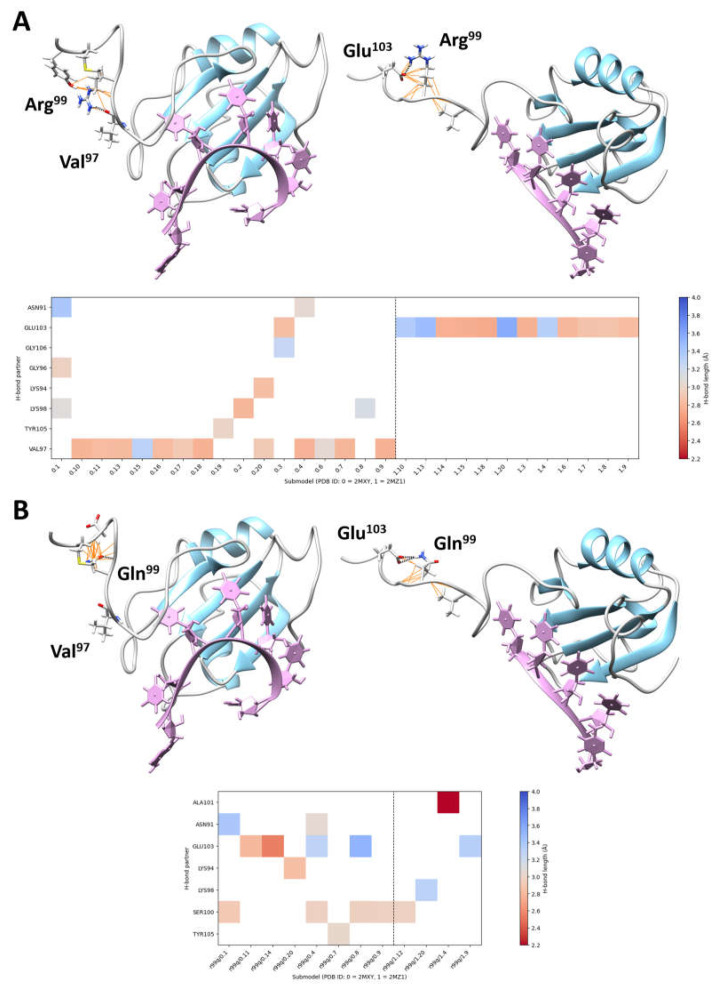
Intramolecular interactions involving Arg^99^ and perturbations introduced by the pathogenic Arg99Gln substitution. (**A**) The 3D structures of the single-stranded RNA-binding motif (RBM) of HNRNPC complexed with RNA consensus sequences (PDB ID 2MXY, submodel 18, 5′-AUUUUUC-3′, left; PDB ID 2MZ1, submodel 9, 5′-UUUUC-3′, right) are shown, with secondary structures and loops colored in sky blue and grey, respectively (**top panel**). The RNA consensus sequences are plum-colored. H-bonds (black dashed lines) and van der Waals bonds (orange lines) involving Arg^99^ are shown. The two most frequent H-bonds observed in the NMR spectrometry models (i.e., involving Val^97^ and Glu^103^) are also indicated (left and right, respectively). The plot (**bottom panel**) shows all the intramolecular H-bonds involving Arg^99^, colored by H-bond length. (**B**) By considering the same 3D structures, the substitution of the arginine residue by a non-charged polar residue, glutamine, abolishes binding to Val^97^ (**left**) and weakens binding to Glu^103^ (**right**) (**top panel**). The plot (**bottom panel**) shows all the intramolecular H-bonds involving Gln^99^, colored by H-bond length.

The structural consequences of the Arg99Gln substitution in HNRNPC folding and function were assessed using the available 3D nuclear magnetic resonance-spectroscopy models (PDB ID: 2MXY, and PDB ID: 2MZ1) [30]. In the generated structure, Arg^99^ forms two key intramolecular interactions with Val^97^ (H-bond, 2.92 Å ± 0.21 SD) and Glu^103^ (salt bridge, 2.94 Å ± 0.27 SD). These bonds contribute to the stabilization and proper folding of the linker region. The replacement of Arg^99^ by Gln was predicted to abolish both the intramolecular H-bond with Val^97^ and electrostatic component of the salt bridge with Glu^103^ (Figure 2).

### 3.3. Assessment of the Clinical Profile Associated with Arg99Gln

We clinically compared the present subject and the previously described individual carrying the same missense change (individual 11) [11]. Their characteristics are summarized in Table 1. Both the individuals shared bilateral cochlear aplasia/hypoplasia and colobomatous microphthalmia as major signs. They also presented with overlapping facial features, including a broad and receding forehead with a high upper hairline, mild synophrys, wave-shaped lids, anteverted nares, a long philtrum, and a thin upper lip with a prominent malar region. Notably, these features were only sporadically reported in the other subjects with *HNRNPC* variants as individual traits [11], indicating that this specific amino acid substitution leads to a unique clinical presentation.

## 4. Discussion

We report the second subject carrying the pathogenic c.296G>A missense variant (Arg99Gln) in *HNRNPC* showing a unique clinical presentation characterized by DD/ID, distinctive facial features, cochlear aplasia, and bilateral colobomatous microphthalmia. The clinical phenotype of the subject fit that of the previously described individual and only partially overlaps with the clinical spectrum of MRD74 [11]. This finding suggests the occurrence of phenotypic heterogeneity involving pathogenic variants in *HNRNPC*.

Haploinsufficiency has been shown to be the pathogenic mechanism underlying the majority of *HNRNPC* variants, affecting proper transcript processing of ID-associated genes. However, the pathological effect exerted by a small number of missense changes remains to be elucidated [11]. Notably, the two individuals carrying the Arg64Trp and the Val108Ile amino acid substitutions, the latter affecting the less abundant major isoform (NP_112604.2) only (the variant is an intronic change, c.317+5G>A, of the processed transcript encoding the short but more abundant HNRNPC isoform; NP_004491.2), presented with a non-specific phenotype with slight dysmorphic features, and peculiar behavior anomalies substantially overlapping with those found in other MRD74 individuals with *HNRNPC* LoF variants. Specifically, these individuals presented with feeding problems (observed in 8/10 individuals with LoF *HNRNPC* variants) and a typical happy demeanor associated with other behavioral anomalies (observed in 7/10 individuals with LoF *HNRNPC* variants). These two clinical signs have not been observed in the two individuals with the Arg99Gln amino acid substitution. On the other hand, excluding the two affected subjects with the Arg99Gln amino acid change, none of the subjects with LP/P variants in *HNRNPC* have been reported to show cochlear aplasia and coloboma. This specific genotype–phenotype correlation suggests the occurrence of a specific consequence of the Arg99Gln amino acid substitution on HNRNPC function.

While the RNA-binding activity of HNRNPC is conferred by the conserved RBM domain, the RNA-binding specificity towards the poly-r(U)_5_ consensus sequence and ability to recognize different transcripts is granted by the linker domain encompassing residues from K94 to M104 [15,16]. Consistently, two HNRNPC truncated mutants, Δ95–290 and Δ105–290, maintained their RNA-binding activity, but lost their high affinity for targets with the poly-r(U)_5_ tract, indiscriminately binding to transcripts with different poly-r(U) consensus sequences (i.e., poly r(U)_8_, poly r(U)_13_) [15]. Within the linker region, Arg^99^, Lys^98^, and Glu^103^ are highly intolerant to any amino acid substitutions, according to the AlphaMissense Pathogenicity Heatmap. Consistently, no substitution affecting Arg^99^ has been reported in public databases (gnomAD v.4.1.0 and *All of Us*). Moreover, only a few benign missense changes have been reported to affect residues within the entire linker domain, indicating that local structural rearrangements of this region may dramatically affect the target recognition process. Structural inspection of the HNRNPC RBM-linker region complexed with the poly-r(U)_5_ stretch showed that the substitution of the positively charged Arg^99^ with Gln, a polar residue, disrupts the salt bridge with the conserved Glu^103^ and abolishes the H-bond with Val^97^. Although functional characterization is not yet available, the structural data indicate that Arg^99^ is a key residue for maintaining the linker domain’s folding and consequently granting specificity in binding to poly-r(U)_5_-containing transcripts.

Cochlear hypo/aplasia and colobomatous microphthalmia are common findings in CHARGE syndrome [32,33,34], which is caused by heterozygous mutations in the chromodomain helicase DNA-binding protein 7 (*CHD7*) gene encoding a chromatin remodeler [35]. Indeed, though the clinical phenotype of the proband did not fulfill the diagnostic criteria of CHARGE syndrome, targeted sequencing and DNAm profiling were performed to exclude this diagnosis. A clinical diagnosis of CHARGE syndrome requires four major criteria or three major criteria and three minor criteria according to Blake et al. [19], with cochlear hypo/aplasia and colobomatous microphthalmia representing major criteria [18,19]. In individuals fulfilling the diagnostic criteria for CHARGE syndrome, *CHD7* sequencing and targeted analyses directed to identify structural rearrangements involving the gene are expected to confirm the diagnosis in all cases. Notably, pathogenic variants in various genes associated with other well-recognized syndromic conditions have been reported in individuals with at least two major signs of CHARGE syndrome, suggesting the occurrence of genetic heterogeneity involving those genes with pathogenic variants specifically associated with phenotypes that should be considered as a differential diagnosis in individuals with an atypical/incomplete CHARGE phenotype [36]. Among them, Kabuki syndrome is a syndromic DD/ID disorder characterized by a distinctive facial gestalt, caused by LoF pathogenic variants in the *KMT2D* and *KDM6A* genes [37]. There is evidence that specific pathogenic variants affecting exons 38 and 39 of *KMT2D* result in a unique phenotype characterized by choanal atresia, hypoplastic nipples, branchial apparatus abnormalities, neck pits, lacrimal duct anomalies, hearing loss, external ear malformations, and thyroid abnormalities, resulting in a condition partially overlapping with CHARGE syndrome, and not resembling Kabuki syndrome [38,39,40]. Similarly, while heterozygous variants in *RERE* cause a neurodevelopmental disorder with or without anomalies of the brain, eye, or heart (NEDBEH, MIM 616975) [41,42], one single recurrent in-frame duplication has been reported in three independent individuals sharing a phenotype characterized by a variable combination of choanal atresia, iris coloboma, abnormal external ears, progressive sensorineural hearing loss with cochlear dysplasia, and ID [36,42]. This finding prompted the authors to propose the inclusion of *RERE* in the genes to be tested in individuals who fulfill the diagnostic criteria for CHARGE syndrome but do not carry pathogenic variants in *CHD7* [42]. The specificity of this genotype–phenotype correlation further highlights the possibility of phenotypic heterogeneity associated with unique gene variants, as observed for the presently identified Arg99Gln substitution in *HNRNPC*. In line with these considerations, a single individual with a clinical diagnosis of CHARGE syndrome, showing a normal chromosomal microarray and negative for intragenic *CHD7* mutations, was identified to carry a missense variant in *EP300*, a gene implicated in Rubinstein–Taybi syndrome 2 (MIM 613684) and Menke–Hennekam syndrome (MIM 618333) [36,43,44].

In conclusion, we provide evidence of a recognizable phenotype associated with the c.296G>A (p.Arg99Gln) change in *HNRNPC* that clinically diverges from MRD74. This phenotype is characterized by DD/ID, distinctive facial features, cochlear aplasia, and bilateral colobomatous microphthalmia. Due to the co-presence of ocular and cochlear involvement, two main features of CHARGE syndrome, we propose that the HNRNPC^Arg99Gln^-related phenotype should be considered as a potential differential diagnosis in subjects with ID and major signs of CHARGE syndrome not fulfilling the minimum criteria for a clinical diagnosis of CHARGE syndrome.

## Figures and Tables

**Table 1:** Clinical features of individuals carrying the c.296G>A substitution in *HNRNPC*.

	Present Study	Individual 11 [11]
Sex	Female	Female
Age at evaluation	14 years	4 years 5 months
*HNRNPC* variant	c.296G>A, p.Arg99Gln (de novo)	c.296G>A, p.Arg99Gln (de novo)
ACMG variant classification	PS2, PM2 (likely pathogenic)	PS2, PM2 (likely pathogenic)
Molecular analysis	WGS (trio)	WGS (trio)
Other clinically relevant variants	None	None
Phenotypic information		
Pregnancy and birth	Normal	Normal, urinary tract infection
Gestational age (weeks)	40	40 + 4
Weight at birth (g)	3730	3540
Length at birth (cm)	51	
OFC at birth (cm)	36	33
Apgar score	8–9	
Growth parameters	18 years	6 years, 8 months
Height (cm)	142.3 cm (−3.0 SD);	122.9 cm (0.7 SD)
Weight (kg)	40.9 kg (−1.8 SD);	29.5 kg (1.7 SD)
OFC (cm)	50 cm (−3.0 SD)	4y5m: 49.3 cm (−1.7 SD)
First words	No words	3 years
Walking	2.5 years	2 years
Language development	No words	Few single words and signs
Gross motor skills	Mild delay	Mild delay
Cognitive deficit	Moderate (IQ 42)	Mild
Seizures	No	No
Hypo-/hypertonia	No	No
Movement disorder	No	No
MRI anomalies	Aplasia of left cochlea and hypoplasia of the right cochlea	Absent cochlea (right), hypoplasia of the left cochlea, absent cochlear nerves
Behavioral anomalies	Stress-related hetero-aggressiveness, stereotypies	None
Sleeping problems	No	No
Facial dysmorphism	Broad and slightly receding forehead, high upper hairline, thick eyebrows, mild synophrys, wave-shaped lids, mild eversion of lower lateral eyelids, narrow and upslanted palpebral fissures, mildly deep-set eyes, deep and long philtrum, sharp nose, anteverted nares, thin upper lip, prominent malar region, preauricular fistula	Broad and slightly receding forehead, high upper hairline, mildly thick eyebrows, mild synophrys, wave-shaped lids, everted lower lateral eyelids, mildly upslanted palpebral fissures, deep-set eyes, smooth and long philtrum, anteverted nares, thin upper lip, prominent malar region, notched lower lateral incisor
Eyes/vision	Bilateral colobomatous microphthalmia	Bilateral colobomatous microphthalmia
Hearing	Sensorineural hearing loss	Sensorineural hearing loss
Cardiac problems	Patent ductus arteriosus	
Renal problems	None	None
Endocrinological problems	Delayed menarche, growth delay, pituitary gland hypoplasia	Premature thelarche
Additional features	Short and broad distal phalanx of the first finger of the hands, hirsutism	Prominent finger pads, hirsute legs, single gray scalp hair

## Data Availability

The sequencing data that support the findings of this work are available on request from the corresponding author. The data are not publicly available due to privacy/ethical restrictions.

## Supplementary Materials

The following supporting information can be downloaded at: https://www.mdpi.com/article/10.3390/genes16020176/s1, Figure S1: DNA methylation profiling analyses; Figure S2: Sanger sequencing chromatograms of the HNRNPC coding region encompassing the c.296G>A variant in the proband.
