# Supplementary material for: The Arg99Gln Substitution in HNRNPC Is Associated with a Distinctive Clinical Phenotype Characterized by Facial Dysmorphism and Ocular and Cochlear Anomalies
Source: Genes (Basel). 2025 Feb 1;16(2):176. doi: 10.3390/genes16020176 (PMC11854916; doi:10.3390/genes16020176)
Supplement: Supplementary file 1 [file genes-16-00176-s001.zip › genes-3427746-supplementary.pdf]

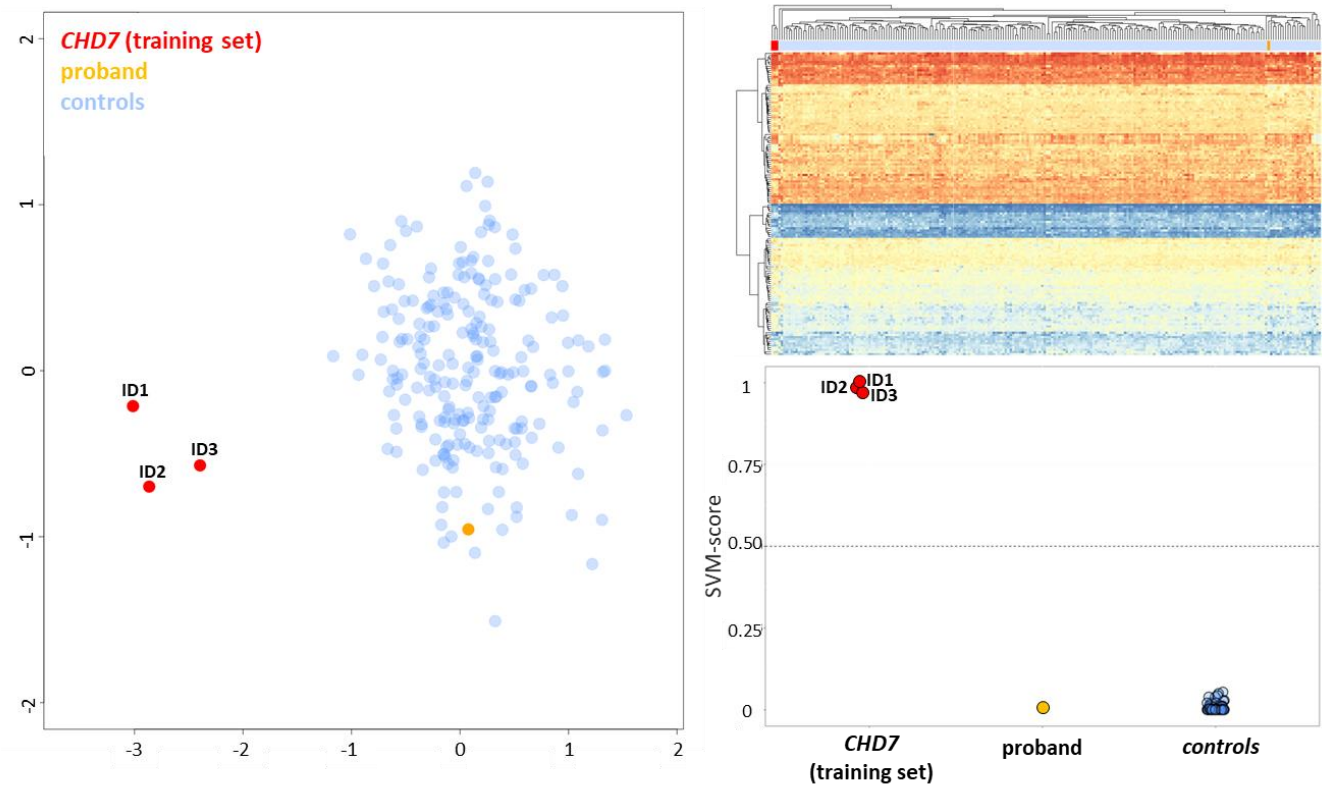

Supplemental Figure S1. DNA methylation profiling analyses. Multidimensional scaling plot (left) and hierarchical clustering (right, top) showing that the peripheral blood (PB)-derived proband's DNA sample (yellow) exhibits a DNAm profie that is similar to controls (light blue) and distinct from three affected individuals with molecularly confirmed clinical diagnosis of CHARGE syndrome (red) (ID1: NM_017780.4:c.2839C>T, p.Arg947Ter; ID2: NM_017780.4:c.7252C>T, p.Arg2418Ter; ID3: NM_017780.4:c.1972G>T, p.Glu658Ter).

The plot reporting the support vector machine (SVM) probability scores from the developed machine learning (ML)-based classifier was trained using the three PB-derived DNA samples from patients with molecularly confirmed clinical diagnosis of CHARGE syndrome. The ML-based classifier refuses a diagnosis of CHARGE syndrome in the proband (yellow) and in 400 in-house controls including healthy individuals and patients with various neurodevelopmental disorders (light blue), with high confidence. SVM-score ranges from 0 (= controls) to 1 (= training set).

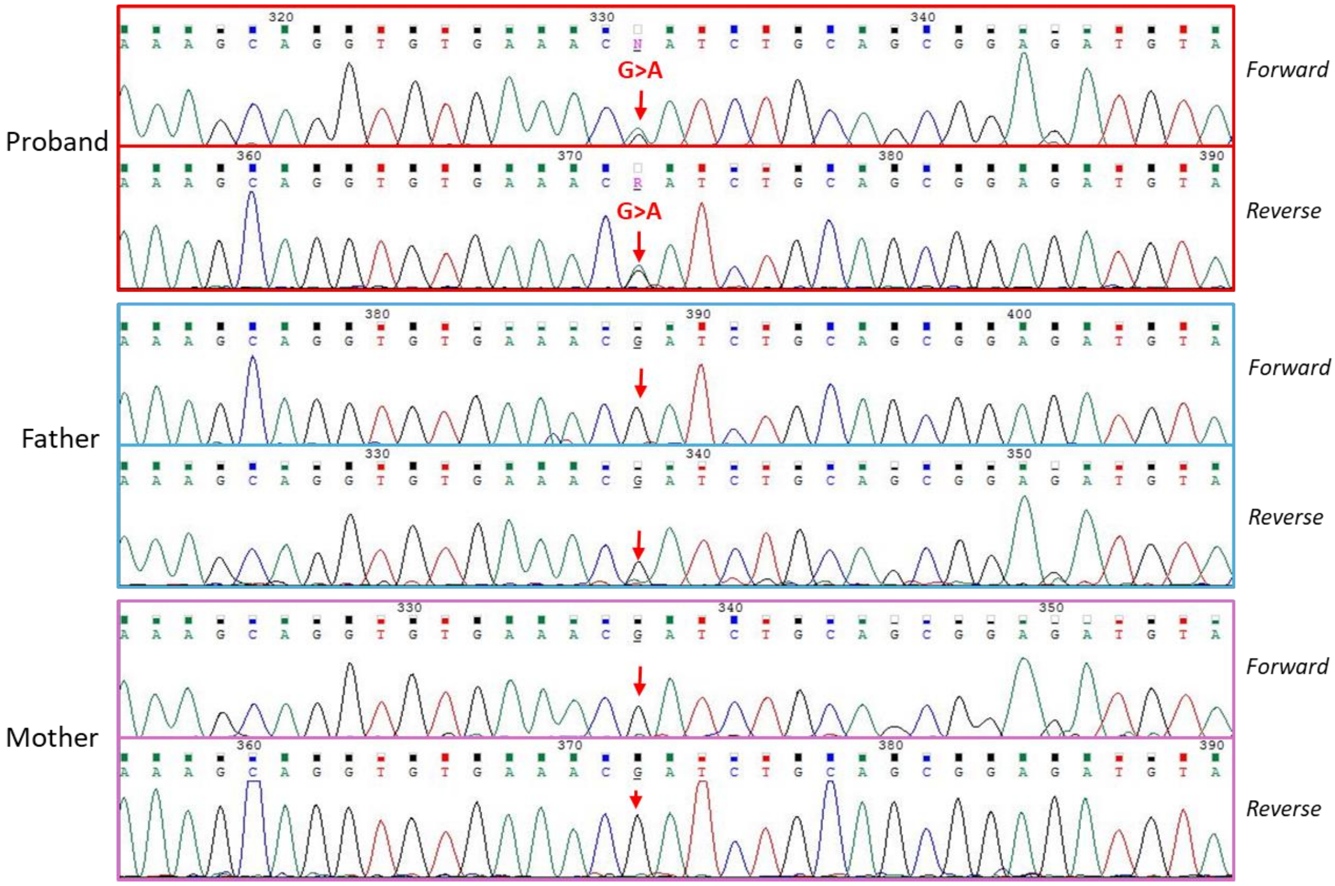

Supplemental Figure S2. Sanger sequencing chromatograms of the *HNRNPC* coding region encompassing the c.296G>A variant in the proband. The proband (red panel) shows the heterozygous G>A transition at cDNA position 296, which is not observed in the father (blue panel) and mother (pink panel), confirming its *de novo* origin.
